# Enhanced immobilization of Prussian blue through hydrogel formation by polymerization of acrylic acid for radioactive cesium adsorption

**DOI:** 10.1038/s41598-019-52600-z

**Published:** 2019-11-08

**Authors:** Daemin Oh, Bokseong Kim, Sungwon Kang, Youngsug Kim, Sungjong Yoo, Sol Kim, Yoonshun Chung, Sungwook Choung, Jeonghee Han, Sunghee Jung, Hyowon Kim, Yuhoon Hwang

**Affiliations:** 1 Korea Institute of Civil Engineering and Building Technology, 283, Goyandae-ro, Ilsanseo-gu, Goyang-si, Gyeonggi-do 10223 Korea; 2 Korea Basic Science Institute, 162, Yeongudanji-ro, Ochang-eup, Cheongju 28119 Korea; 3 Korea Atomic Energy Research Institute, 111, Daedeok-daero 989Beon-gil, Yuseong-gu, Daejeon 34057 Korea; 4 Seoul National University of Science and Technology, 232 Gongneung-ro, Nowon-gu, Seoul 01811 Korea

**Keywords:** Materials science, Materials chemistry, Polymer chemistry

## Abstract

In this study, a hydrogel impregnated with powder activated carbon (PAC), MAA-PAC, was synthesized through the polymerization of acrylic acid (AA) and PB was immobilized using the carboxyl group of AA. In this process, an adsorbent with an enhancement of PB content and stability of immobilization was developed through the additional supply of Fe^3+^ ions by the layer by layer (LBL) assembly. XRD, FT-IR, SEM (EDS), TEM (EDS, mapping), and TG analyzes of the LBL and non-LBL groups were performed to confirm the change of PB content in the adsorbent as the LBL assembly was applied. The stability of PB immobilization was confirmed during the washing process after the synthesis of the adsorbent. When the LBL assembly process was applied as a PB immobilization strategy, the PB content in the adsorbent was improved and PB leakage was not observed during the washing process. The maximum adsorption (q_m_) for cesium in the MAA-PAC-PB LBL group that showed high PB content was 40.03 mg/g, and the adsorption isotherm was more suitable for the Langmuir model than the Freundlich model. The LBL group showed a high removal efficiency of 99.81% and a high DF value (525.88) for radioactive cesium (120 Bq/g). These results demonstrate the potential efficiency of the MAA-PAC-PB LBL group for the decontamination of radioactive cesium-contaminated water systems. Furthermore, it was verified that the LBL group of MAA-PAC-PB could be used as an adsorbent without an additional design of the existing water treatment facility. This can an economical decontamination method for removing radioactive cesium.

## Introduction

Nuclear power generation, which is an effective source of energy and electricity production, is a core technology for electricity production^1^. In the long run, nuclear power generation can produce energy and electricity even when additional energy sources, such as wind and solar heat, are not available due to bad weather conditions. Furthermore, the total cost of electricity production is much lower than other energy sources, such as fossil fuels and renewable energy sources^2^. For this reason, some countries are investing in R&D to increase the reliability of energy production systems by nuclear power^3^. However, the operation of a nuclear power plant may cause a large-scale disaster-related nuclear power plant accident, such as Fukushima and Chernobyl accidents. Recently, an earthquake has occurred near a nuclear power plant operating in the southeast region of the Republic of Korea, and this raises a concern about the large-scale nuclear accident that was mentioned previously^4^. In 2011, a large number of radioactive nuclear species were introduced into the atmosphere and sea through the Fukushima nuclear accident in Japan, which is still happening^5^. The spread of radioactive cesium due to a nuclear accident can lead to large-scale environmental pollution. Compared to other radioactive contaminants, cesium is characterized by its ability to dissolve readily in water, high gamma-ray emission, and a half-life of 30 years^6^. Therefore, cesium is likely to contaminate water and soil as it is spread in the aerosol form due to radioactivity fallout during a nuclear accident. It is very difficult to remove cesium from water because it has a small hydration radius and a higher diffusion coefficient than other radioactive contaminants^7^. Although studies have been conducted on ion exchange^8^, membrane filtration^9^, and reverse osmosis^10^ to remove radioactive cesium that is diffused into the water system to date, it still requires installation in an existing facility and a new operation. Therefore, it is required to develop an efficient and economical water treatment technique for removing radioactive cesium in water. Adsorption is one of the simplest processes for removing radioactive cesium diffused in the water, and therefore, it is considered as an economical method to effectively and quickly remove the cesium since no additional treatment is required after the adsorption process for the target contaminants^11,12^.

Prussian blue (PB) is a blue dye and is synthesized by reacting iron(III) chloride (FeCl_3_) with potassium ferrocyanide (K_4_[Fe(CN)_6_)^13^. PB is a type of metal hexacyanoferrate (MHCF), a cubic lattice structure formed of cyanide and metal ions. It is known that selective adsorption of dissolved cesium in water is possible^13,14^. Mechanisms for Cs adsorption by MHCF perform mainly through ion exchange of Cs^+^ and K^+^, and percolation of Cs^+^ by vacancies site from the surface, proton exchange with Cs^+^. The selectivity Cs adsorption of PB was also explained by the complete dehydration of alkali cations^15^. However, PB is difficult to recover after the adsorption process of cesium because nanomaterial with a particle size of less than 200 nm and highly dispersible in water^16–18^. Such characteristics of the PB are disadvantageous for direct application to existing water treatment systems for treating cesium-contaminated water. Various studies have been conducted including the capture adsorbent^19,20^, which is impregnated with PB powders to immobilize the PB for cesium removal, and the inducing adsorbent^21,22^, which induces PB immobilization through *in-situ* chemical bonding by specific functional groups. Recently, Wi *et al*. suggested the use of acrylic acid for surface modification of supporting materials (polyvinyl alcohol sponge, cellulose filter) in order to form carboxylic acid groups. The carboxylic acid group provided better stability of PB resulting less leaching into water. Moreover, they suggested a layer-by-layer (LBL) assembly process as an efficient method for PB immobilization to increase the PB content in the adsorbent^23,24^.

The hydrogel is a cross-linked hydrophilic polymer that typically expands in water. The network structure in the hydrogel may be formed by chemical cross-linking or physical cross-linking. Such hydrogels are generally prepared via free radical polymerization mechanisms, in which the monomer units are linked in long chains through a double bond and a cross-linking reagent^25–27^. Hydrogel that can efficiently absorb moisture has a polymer network with dissociated ionic functional groups. Recently developed acrylic acid, which is one of the synthetic materials for the hydrogel, has been studied in a wide range of fields because it is inexpensive and easily polymerized with molecular weight polymers^28,29^. The hydrogel network that is formed by acrylic acid (AA) has an ability to absorb water multiple times depending on the weight in water and forms the basis of a substance called superabsorbent^30^. A study on the adsorption of heavy metal ions using the carboxyl group, which is an acidic functional group of the hydrogel synthesized through the polymerization reaction of polyacrylic acid, was conducted^31^. However, since the mechanical strength is weak and it has limited applications, studies on of the hydrogel synthesis have been conducted to improve material stability by impregnating activated carbon^32^.

The purpose of this study is to develop an adsorbent material that can be applied immediately in the existing water purification plant without any additional facility for the water source contaminated by cesium. First, a hydrogel was synthesized through the polymerization of AA, which is used as a heavy metal adsorbent. In order to improve the mechanical strength, powder activated carbon (PAC) was impregnated during the synthesis process of the hydrogel. We named this material as MAA-PAC (Modified acrylic acid-PAC). The carboxyl group, which is a functional group of AA, is a strong Lewis base and forms bonds with Lewis acid along with heavy metal ions^33^. Through this process, Fe^3+^ ions, which are one of the synthetic materials of the PB, were adsorbed with the MAA-PAC first. After that, the PB in the MAA-PAC particle was reacted with potassium ferrocyanide to induce the synthesis through the *in-situ* method. Finally, the MAA-PAC-PB was synthesized by the additional reaction with Fe^3+^ ions for growth and stable immobilization of the PB particles through the LBL assembly process. This is also a synthetic method for controlling the PB outflow. The characteristic analysis was conducted to analyze the PB content in the adsorbent through the LBL assembly process and the non-LBL group of MAA-PAC-PB that was prepared by the conventional PB synthesis method. The adsorption performances of synthesized adsorbents on radioactive cesium (Cs-137) and isotope cesium (Cs-133) are evaluated.

## Results and Discussion

### Optimization of MAA-PAC synthesis

In the conventional method, the Prussian blue (PB) is made by synthesizing iron (III) chloride and potassium ferrocyanide. Evaluation of the adsorption efficiency of Fe^3+^ ions in the *in-situ* synthesis of the Prussian blue, which is preferentially proceeded for the synthesis of the MAA-PAC-PB (non-LBL group), is also a step to predict the content of the PB that is present in the MAA-PAC particles. Therefore, in this synthesis process, Fe^3+^ adsorption performance of the MAA-PACs that are finally synthesized by injecting different concentrations of sodium hydroxide for polymerization of AA was evaluated in advance. Among the injected acrylic acid solutions during the synthesis process of MAA-PAC, sodium hydroxide was injected at various concentrations. The degree of neutralization during the synthesis process of the hydrogel, which is a water-absorbing polymer, was checked to confirm the effect of the change of the water absorption rate on the reaction with the target contaminant. When acrylic acid is neutralized with sodium hydroxide, the carboxyl group, which is negatively charged and attached to the polymer network, causes electrostatic repulsion that extends the network of hydrogels. Within a certain range of the degree of neutralization, the electrostatic repulsive force increases as the degree of neutralization increases, which increases the absorptiveness. However, when the degree of neutralization is further increased, more Na^+^ ions are generated to screen negative charges of the carboxyl group. Therefore, the electrostatic repulsion the adsorption efficiency is reduced^34^. Prussian blue was synthesized by mixing iron(III) chloride with potassium ferrocyanide^35,36^. This *in-situ* synthesis method has been used to efficiently form PB particles inside the pores of the porous support. In this study, the Prussian blue was immobilized by using a carboxyl group, which is a functional group of the MAA-PAC. It was possible to indirectly determine the amount of the Prussian blue, which is immobilized according to the amount of adsorption after Fe^3+^ ions are reacted with iron(III) chloride to be absorbed. As shown in Table 1, the adsorption efficiency of Fe^3+^ ions of the MAA-PAC, which was synthesized according to the degree of neutralization, was highest when sodium hydroxide was injected at a concentration of 1 M, and this result is similar to the results of the previous studies^30^. Characteristic analysis of MAA-PAC-PB, which is an adsorbent where the synthesized Prussian blue is immobilized in the same conditions, and the cesium adsorption characteristics were conducted.

**Table 1:** Evaluation of Fe^3+^ ion adsorption efficiency of MAA-PAC by the concentration of sodium hydroxide.

**Sodium hydroxide conc. (M)**	0.01	0.1	1	2
**Fe** ^**3+**^ **adsorbed amount (mg/g)**	166.7	384.1	476.7	340.2

### Effect of LBL assembly on stable PB synthesis

#### Change of functional group during MAA-PAC-PB synthesis(FT-IR and XRD

PB has been studied as suitable electrode materials for battery or sensor. The LBL techniques for the PB formation are well-known in the fabrication of PB into a thin film. For example, Pyrasch *et al*. described a method of fabricating PB films by repeated immersion of pretreated quartz or ITO coated glass into potassium hexacyanoferrate and ammonium ferrous sulfate solution for the production of PB ultra-thin membranes^37^. Jin *et al*. proposed a method of synthesizing PB-coated membranes by repeatedly reacting a solution prepared with potassium ferrocyanide, potassium chloride or Iron (III) chloride, potassium chloride on a pretreated substrate^38^. However, the LBL techniques were not well adapted in the field of cesium adsorption. In this study, the LBL technique was applied for production of Cs adsorbent in order to enhance overall stability of PB in the immobilization. As mentioned above, LBL assembly of PB was performed by sequentially reacting iron (III) chloride, potassium ferrocyanide, and Iron (III) chloride with the hydrogel type MAA-PAC.

**Figure 1:**
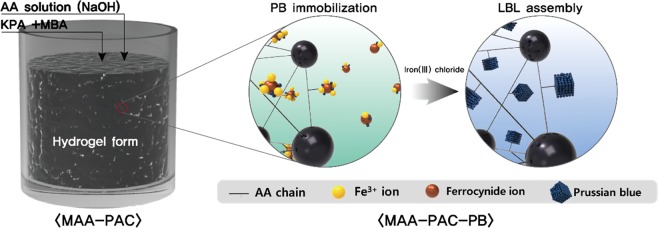
Scheme for the hydrogel by polymerization of acrylic acid and immobilization of Prussian blue by LBL assembly.

**Figure 2:**
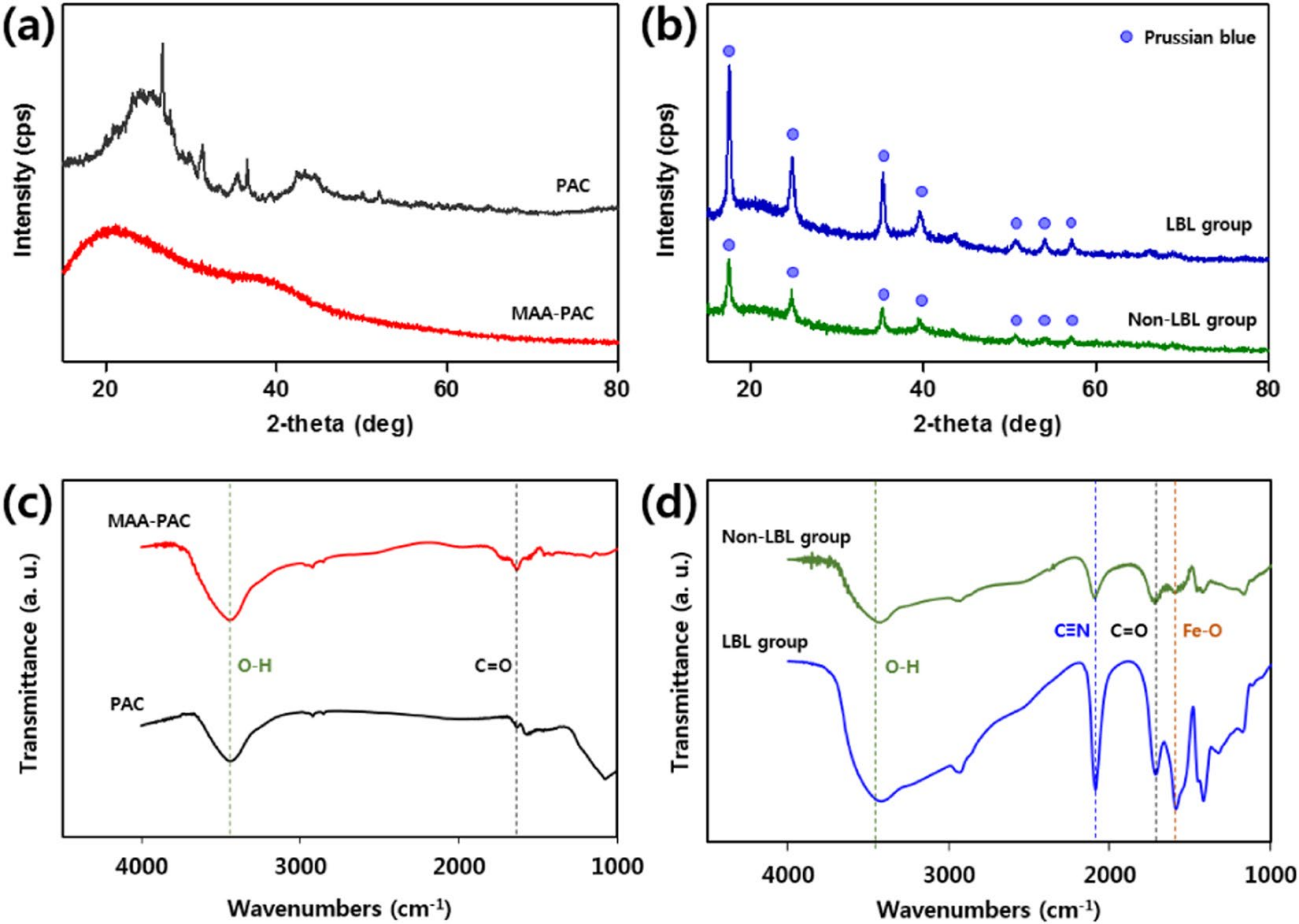
Characterization of synthesis steps materials of MAA-PAC-PB. (**a**,**b**) XRD pattern, (**c**,**d**) FT-IR spectra, PAC(black), MAA-PAC(red), MAA-PAC-PB non-LBL group(green), MAA-PAC-PB LBL group(blue).

As shown in Fig. 1, Prussian blue was immobilized through the *in-situ* method, which is the general PB synthesis method, after the synthesis of the MAA-PAC. FT-IR and XRD analyses were conducted to observe changes in adsorbent properties during the immobilization of the Prussian blue, and the results are shown in Fig. 2. Figure 2a shows the XRD pattern of PAC and MAA-PAC. The characteristic peaks of PAC showed broad peaks near 26 and 44 degrees, which is known to show the amorphous nature of activated carbon^39^. In the pattern of MAA-PAC, there was not a distinct peak when compared to PAC. This indicates that MAA-PAC is an amorphous compound. Figure 2b shows the XRD patterns of the LBL and non-LBL groups of MAA-PAC-PB. As a result of the XRD analysis, the characteristic peaks of the Prussian blue (17.5, 24.8, 35.4, 39.8, 50.46, 54.26, 57.4 degrees), which were not observed in the PAC and MAA-PAC analyses, were found in the analysis of the non-LBL and LBL group^40,41^. Through the FT-IR analysis of the MAA-PAC, a peak was found due to the C=O bond in the functional group of the carboxyl group, which is the functional group of the hydrogel formed through the polymerization reaction of acrylic acid, at around 1,615 cm^−1^ (Fig. 2c). In the MAA-PAC-PB analysis of the non-LBL and LBL group, a new absorption peak that corresponds to the stretching vibration of the cyanide group (C≡N), which generally exhibits the Prussian blue characteristic at around 2,081 cm^−1^, was observed(Fig. 2d). Through this observation, the presence of Prussian blue of the non-LBL group was confirmed in the particles^42^. Furthermore, a characteristic peak for adsorbed Fe ions, which appeared in the Prussian blue immobilization process through the *in-situ* synthesis, was observed, and this confirmed the characteristic change. These results suggest that LBL assembly is a more effective synthesis method for the formation of the PB particles in the *in-situ* synthesis of the PB using carboxyl groups^23^.

#### Distribution of PB(SEM/TEM)

**Figure 3:**
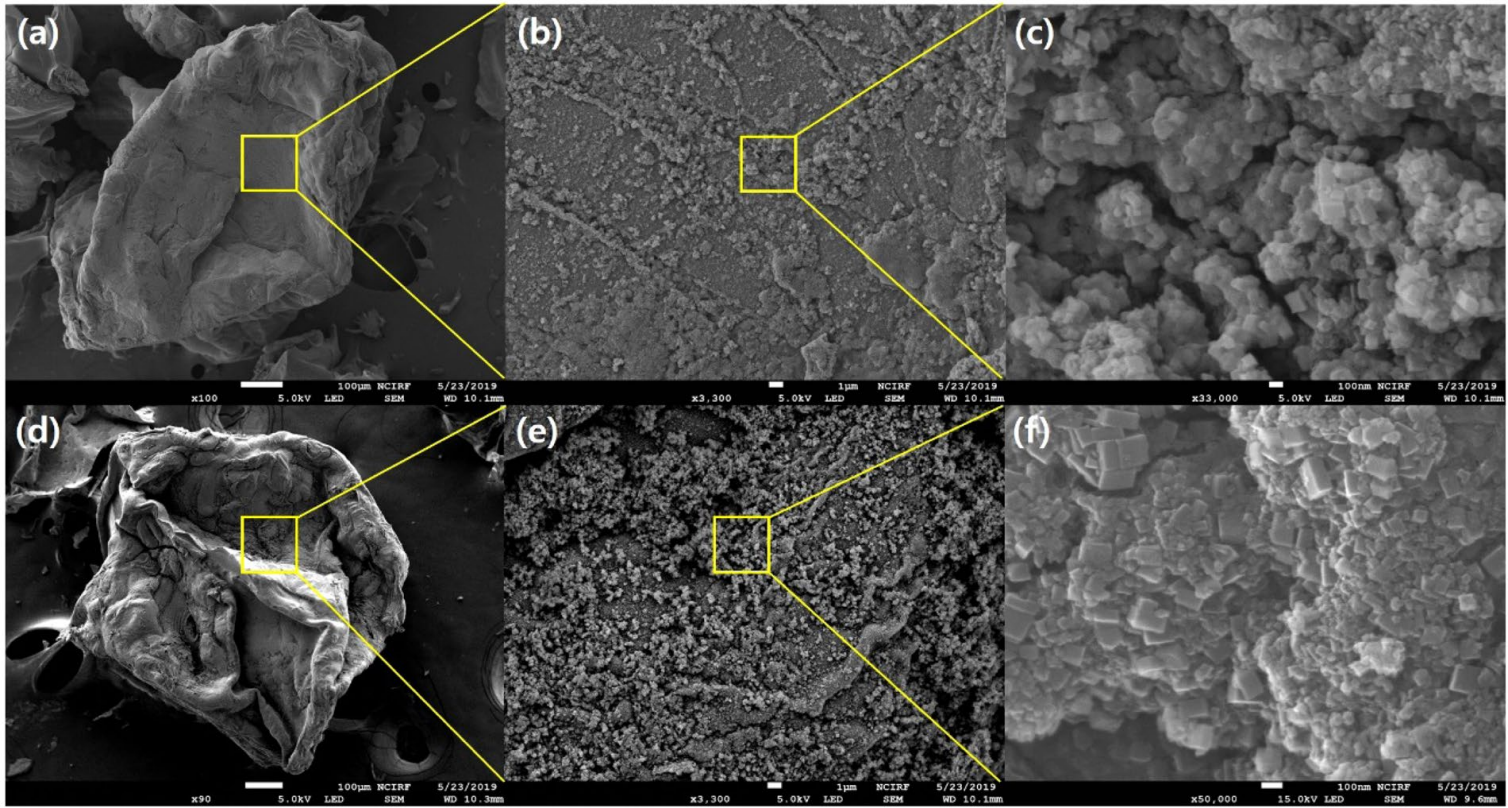
SEM image of MAA-PAC-PB, (**a,b,c**) Non-LBL group, (**d,e,f**) LBL group.

The SEM-EDS analysis was performed to further investigate by comparing it to the analysis results presented above. Figure 3 shows the characteristic morphology of the MAA-PAC-PB non-LBL and the LBL group through the SEM image. The SEM image of the MAA-PAC after drying and pulverization of hydrogel, which was prepared by the polymerization reaction of acrylic acid impregnated with PAC, is shown in Fig. S1. The MAA-PAC before the PB synthesis appears as a translucent crystalline form of the compound as moisture in the hydrogel is removed during the drying process. Similarly, the particles of the MAA-PAC were also found to be crystalline. It was found that the rough part in the image was due to the grinder blade in the grinding process. Figure 3a shows an SEM image of the MAA-PAC-PB non-LBL group adsorbent material. Compared with the MAA-PAC, the surface of the particle was found to be somewhat rough. This is due to the change in the synthesis process of the PB, and the SEM image at 3300x magnification confirmed the PB particles distributed indiscriminately. Furthermore, in the SEM image at 10000x magnification, it was confirmed that a lattice-shaped PB particle was formed in each part^43,44^. Unlike the MAA-PAC, the surface of non-LBL group particles of the MAA-PAC-PB showed irregular bending. This is due to the swelling of the MAA-PAC particles in water through the PB immobilization and *in-situ* synthesis method, which changed the overall particle surface after the drying process^45^. Figure 3d shows the SEM image of the MAA-PAC-PB after the LBL assembly process. As with the particles of the non-LBL group of the MAA-PAC-PB, irregular bending appeared on the particle surface. However, as shown in Fig. 3e, the distribution of the PB particles that are immobilized on the particle surface of the LBL group was visually confirmed to be wider. The SEM images at 10,000x magnification showed that the lattice-shaped PB particles were more dense and clear compared to the non-LBL group. This result is similar to the results of FT-IR and XRD analyses, and it is probably because the lattice-shaped PB particles were grown much larger as Fe ions and additional reaction steps were performed in the LBL assembly process^23^.

**Figure 4:**
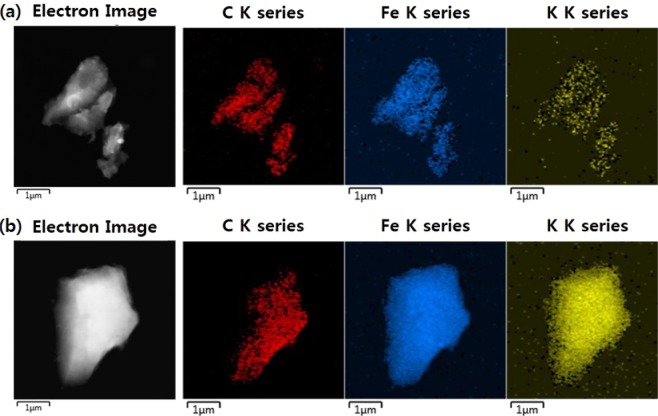
TEM-EDS mapping image of MAA-PAC-PB, (**a**) non-LBL group, (**b**) LBL group.

The SEM-EDS analysis also supported the SEM image observation. First, the EDS analysis results of the MAA-PAC, which is the initial material before the PB synthesis, were confirmed through Table S1, and 97.23% and 2.77% of C and O elements were contained, respectively. In comparison, Fe and K were newly observed in EDS analysis of the non-LBL group of the MAA-PAC-PB. It was concluded that this was caused by the reaction of iron(III) chloride with potassium ferrocyanide during the immobilization of the PB. Compared with the non-LBL group, the content of Fe and K in the LBL group increased by a factor of three and a factor of two, respectively. These results were further confirmed by the TEM-EDS mapping analysis of the LBL group and the non-LBL group as shown in Fig. 4. Both the non-LBL group and the LBL group contained C in the surface of the particles because they went through the PB immobilization with the MAA-PAC as the support. Both adsorbents contained Fe and K elements, which were derived from iron(III) chloride and potassium ferrocyanide during the PB synthesis, respectively^16^. The LBL group has significantly higher ratios of Fe and K than the non-LBL group does. The result of the TEM-EDS analysis(Table. S2) was found to be similar to that of the SEM-EDS analysis as shown in Table 2. The Fe element content of the LBL group was about 7 times higher, and the K element content was about 6 times higher than those of the non-LBL group in the LBL group. This result showed that the PB particles were successfully grown in the MAA-PAC by the additionally reacted Fe in the LBL assembly process, and the immobilization of the PB through this would be more efficient and stable^23^.

**Table 2:** SEM-EDS analysis results of MAA-PAC-PB(non-LBL group), MAA-PAC-PB(LBL group).

Items	Non-LBL group		LBL group	
Elements	Weight %	Atomic %	Weight %	Atomic %
C	64.47	77.26	32.80	66.76
O	21.05	18.94	1.72	2.62
K	0.65	0.24	10.39	6.50
Fe	13.83	3.56	55.09	24.12
Totals	100		100	

#### PB content(TGA)

**Figure 5:**
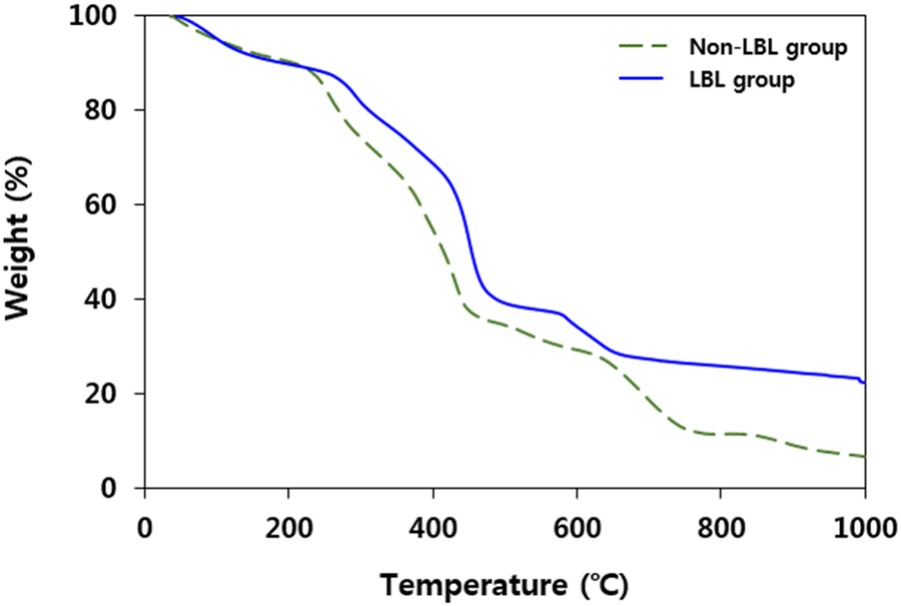
Thermogravimetric(TG) analysis result of MAA-PAC-PB LBL group and non-LBL group.

Figure 5 shows the TGA results of the MAA-PAC-PB non-LBL group and the LBL group that were performed at a heating rate of 10 °C/min under an inert atmosphere of nitrogen. The non-LBL group showed a mass loss of about 11.5% until it reached 220 °C, which is considered to be a loss of water that remained in the adsorbent material. After that, a gradual loss of mass was observed up to 400 °C as the temperature increases, which corresponds to the loss of oxygen functional groups of the acrylic acid network and the PB that constitutes the MAA-PAC. (3d adsorbent paper) The weight loss up to 680 °C was considered to be due to the decomposition of the cyanation group of the PB. It has been reported that the PB particles can be decomposed by the collapse of the bond between Fe(II, III) and the cyanide group as C≡N is converted to N_2_O and CO_2_^30^. Therefore, the residual component after the TG analysis could be Fe_2_O_3_. The weight loss of the adsorbent material was then confirmed to be due to pyrolysis of the carbon skeleton of the impregnated PAC^46^. For the non-LBL group, a TGA curve, which is similar to that in the LBL group, was observed. The mass loss of 12.7% by residual water was observed until it reached 220 °C, the mass loss by loss of oxygen functional group of acrylic acid and PB was observed up to 380 °C, and the mass loss by the decomposition of the cyano group of the PB group was observed up to 720 °C. Overall, the residual masses of 6.94% and 22.41% were observed in the non-LBL group and the LBL group, respectively. From the residual material after the TG analysis, the calculated Fe contents were 4.85 and 15.7%, respectively. The content of the PB was calculated as 10.2% for the non-LBL group and 34.2% for the LBL group.

#### Immobilization stability of PB during washing

**Figure 6:**
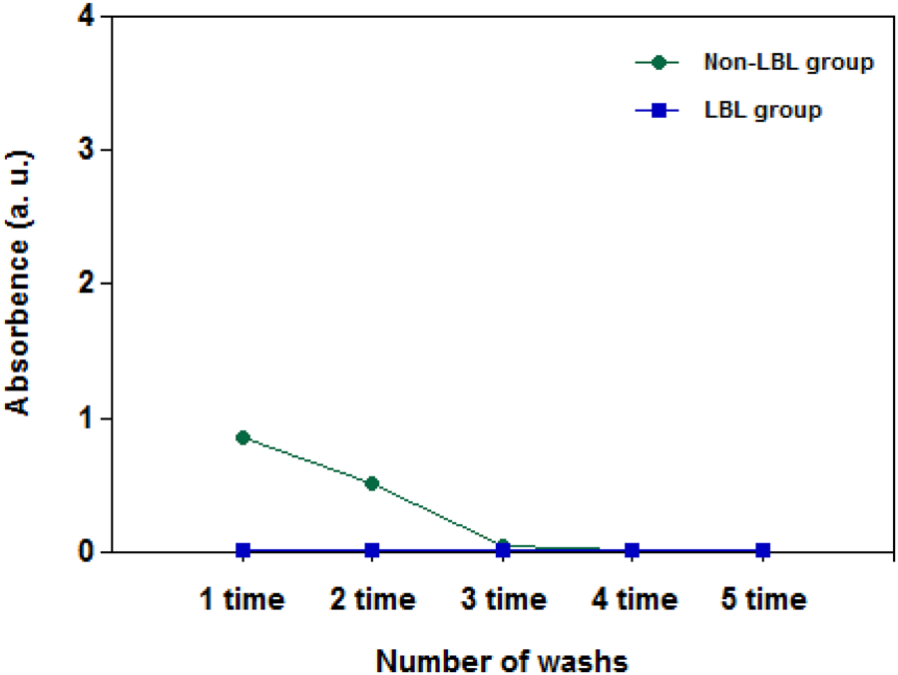
PB elution after being synthesized LBL and non-LBL groups of MAA-PAC-PB by deionized water.

Figure 6 shows the results of the UV-vis analysis of the washing water to analyze the PB outflow after the PB immobilization of the non-LBL group and the LBL group of the MAA-PAC-PB. In order to evaluate the structural stability of the adsorbent, which was synthesized through the two PB immobilization processes presented in this study, the PB was synthesized and washed with distilled water to analyze the washing water in the range of 690 nm of the spectrophotometer. For the LBL group, the total absorbance was found to be in the range of 0.01 in the washing water analysis after the water was stirred for up to 5 times (10 hr). It was concluded that the LBL assembly process resulted in stable immobilization of the PB. It was also considered as data that could support the results of the previous analysis. On the other hand, in the washing water analysis of the non-LBL group that was synthesized through the sequential reaction between iron(III) chloride and potassium ferrocyanide, which is a conventional PB synthesis method, it was confirmed that a small amount of PB leaked from the adsorbent material during the first and second washings, but no PB leakage was observed from the third to the fifth washings. In the decontamination of cesium-contaminated water using the LBL group, secondary water contamination due to the outflow of the immobilized PB in the adsorbent would not be observed.

#### LBL assembly for stable PB immobilization

As mentioned above, in various studies, PB was synthesized by an *in-situ* synthesis method to immobilize PB particles in the support. However, if an appropriate concentration of the Fe^3+^ ion, which is a precursor in the PB synthesis in the conventional method, is not formed, the residue will remain in the support. Likewise, the conventional *in-situ* synthesis method makes the PB synthesis process unstable during the coating process of the ferrocyanide compound due to insufficient Fe^3+^ ions^23^. On the other hand, the synthesis method through the LBL assembly process can achieve stable immobilization of PB particles due to the additional supply of Fe^3+^ ions. As a result, this will lead to the growth of the PB particles and improve the cesium adsorption performance. In this study, a hydrogel, which is synthesized through polymerization of AA, was used as a support. This is because AA has a carboxyl group as a functional group. Since the carboxyl group is a strong Lewis base, it forms strong bonds with Lewis acids along with heavy metal ions. This has been studied as an adsorbent material for adsorbing cadmium, which is one of the heavy metals^33^. We used the functional group of the AA. After the Fe^3+^ ion, which is one of the synthesis materials of the PB, was preferentially adsorbed using the carboxyl group of AA and reacted with ferrocyanide ions. After that, the additional supply of Fe^3+^ ions stabilized the immobilization of the PB, which is synthesized in the MAA-PAC particles, through which the outflow of the PB in the adsorption process was tried to be controlled. Furthermore, the cesium adsorption efficiency of the MAA-PAC-PB was tried to be improved through the growth of PB particles.

#### Cesium adsorption performance test using Isotope(Cs-133)

First of all, the cesium adsorption test was performed to investigate the effect of MAA-PAC on the cesium adsorption. Cesium removal efficiency of MAA-PAC, MAA-PAC-PB, and PB were tested by 110 mg/L of cesium solution and 0.2 g/L of adsorbent dosage. MAA-PAC showed only 3.84% of removal rate, while 74.5% and 90.65% of removal rate was shown for MAA-PAC-PB and PB, respectively (Fig. S2). In this result, it can be concluded that the adsorption performance of MAA-PAC-PB almost came from PB, not from MAA-PAC. Isothermal adsorption experiments were conducted to evaluate the cesium adsorption performance of the MAA-PAC-PB LBL group, where the stable immobilization was confirmed by PB leaching test. Isothermal adsorption models are widely used to confirm the progress of the adsorption process of the adsorbent and to investigate the adsorption mechanism. These adsorption isotherms are fundamental requirements for adsorption system design. The adsorption equilibrium data can be used to know the adsorbent capacity that is necessary to remove the unit mass of the contaminant. The Langmuir isotherm model represents a single layer adsorption process of cesium on a uniform adsorbent surface. The Langmuir equation is shown in Eq. (1).

1 $$\frac{1}{{q}_{e}}=\frac{1}{b{C}_{e}{q}_{m}}+\frac{1}{{q}_{m}}$$

where q_e_ and q_m_ indicate the equilibrium adsorption capacity and the maximum adsorption capacity, respectively, b is the Langmuir constant associated with the adsorption energy. The q_m_ and b values can be calculated from the intercept and slope of 1/q_e_ to 1/C_e_. The Freundlich isotherm is given by Eq. (2), assuming that the surface of the adsorbent has a spectrum of active sites with different adsorption energies. In this equation, K_f_ can be defined as the adsorption or partition coefficient and it indicates the amount of cesium adsorbed on the MAA-PAC-PB LBL group for a single equilibrium concentration. The slope 1/n is in the range between 0 and 1 and is a measure of the adsorption strength or surface heterogeneity. n is a constant that indicates the adsorption strength^47^.

2 $${q}_{e}={K}_{f}{{C}_{e}}^{1/n}$$

**Figure 7:**
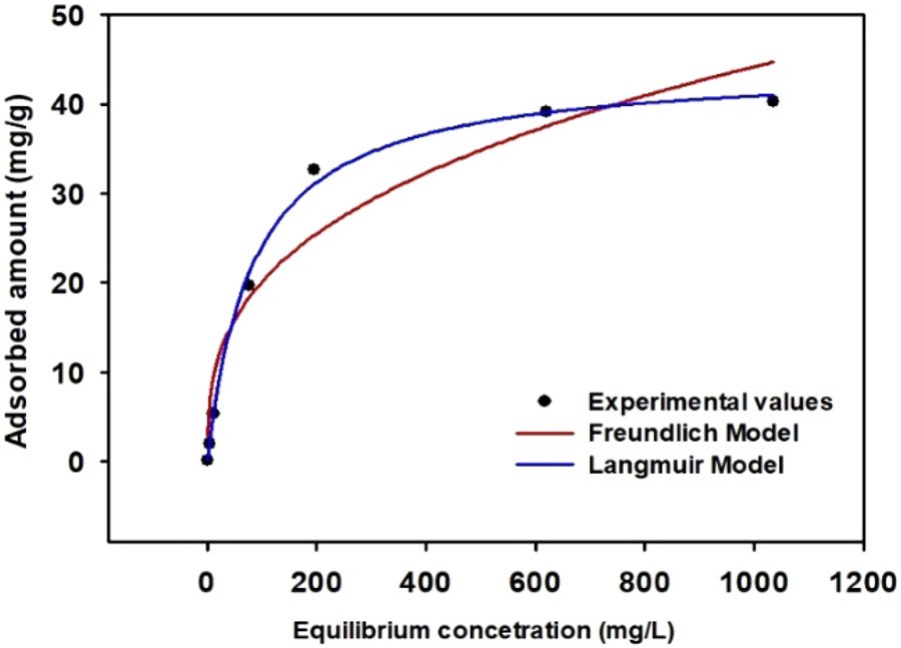
Cesium adsorption isotherm of MAA-PAC-PB LBL group.

The adsorption capacity was increased rapidly as the initial concentration of Cs was increased. This is probably dues to the existence of an active site that is favorable for the Cs adsorption of the adsorbent^22^. The Langmuir model and the Freundlich model constants of the LBL group are shown in Table 3. The correlation coefficient (R^2^) of the Langmuir adsorption model was 0.9971, and this is higher than the correlation coefficient (R^2^) of the Freundlich adsorption model, which is 0.9332. It was concluded that the adsorption behavior of Cs was uniformly adsorbed to the single-layer on the surface. The maximum adsorption (q_m_) of the LBL-group was 40.03 mg/g (Fig. 7)^47^. The result obtained from our study is in the highest range of qm value for structured adsorbents containing PB (Table S3).

**Table 3:** Isotherm parameters for the Cs-133 adsorption onto MAA-PAC-PB LBL group.

Langmuir isotherm			Freundlich isotherm		
q_max_ (mg/g)	b (L/mg)	R^2^	K_f_ (mg^1−1/n^·L^1/n^/g)	1/n	R^2^
40.03	0.0119	0.9971	4.1568	0.3422	0.9332

**Figure 8:**
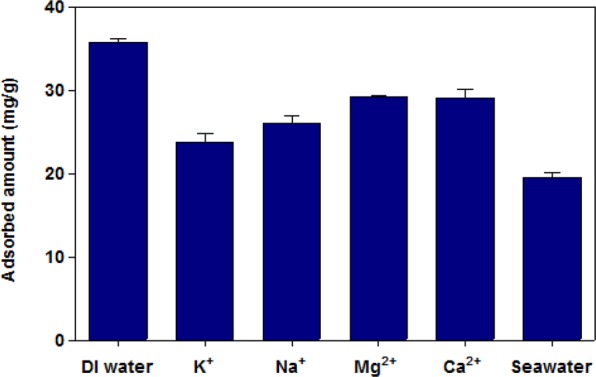
Adsorption of Cs-133 compared to competitor cations such as Na^+^, K^+^, Ca^2+^, Mg^2+^.

Adsorption experiments were conducted in a solution, in which competitive ions such as K^+^, Na^+^, Ca^2+^, and Mg^2+^ exist, to evaluate the selective adsorption efficiency of the MAA-PAC-PB on Cs. A solution with 100 mg/L Cs and 3,000 ppm of competitive ion was prepared and reacted with 0.01 g of the MAA-PAC-PB LBL group. As shown in Fig. 8, when the q_e_ value of distilled water with competitive ions was compared with that of distilled water without competitive ion, the q_e_ value of distilled water with competitive ions tended to decrease. The q_e_ value was the lowest in the presence of K^+^ ion, but the decrease of q_e_ value was less than 40% even though the K^+^ concentration was 40 times higher than Cs^+^. This high selectivity toward Cs^+^ can be explained based on the mechanism of Cs-adsorption by PB. It was reported that Cs-adsorption by PB was governed by three mechanisms, (a) mainly ion-exchange between Cs^+^ and K^+^, (b) percolation of Cs^+^ cations through vacancy sites from the surface, and (c) proton-exchange with Cs^+^. The excellent selectivity of Cs was mainly due to the lowest hydration energy of Cs^+^, leading to the ion exchange^15^. The hydration energy of tested cations (Cs^+^, K^+^, Na^+^, Mg^2+^, Ca^2+^) was reported as Cs^+^ (−264 kJ/mol), K^+^ (−320 kJ/mol), Na^+^ (−406 kJ/mol), Mg^2+^ (−1926 kJ/mol), Ca^2+^ (−1579 kJ/mol), respectively^48^. The experimental results described above were very consistent with previous reports regarding the issue of selectivity^20^.

In order to confirm the ion exchange between K^+^ and Cs, an additional experiment was conducted (Table S4). The initial concentration of K^+^ and Cs^+^ was 407.5, 109.6 mg/L, respectively. The final concentration K^+^ and Cs^+^ after the adsorption experiment 423.3, 57.35 mg/L, respectively. Therefore, the variation of K^+^ and Cs^+^ concentration was 0.40 mM and 0.32 mM, and these values are quite similar. This result clearly indicates the Cs^+^ removal mechanism by PB. K^+^ ion exists in the lattice structure of PB, and it was exchanged by Cs^+^ due to the different hydration energy.

In addition, an extreme environment similar to seawater was established, and the change of the Cs removal performance of the LBL group was observed. As a result, the adsorption performance was degraded, but the q_e_ value was about 20 mg/g in seawater as well.

#### Decontamination of radioactivity cesium(Cs-137)

Experiments were conducted to evaluate the removal efficiency of radioactive cesium in the MAA-PAC-PB LBL group. The adsorbent of the LBL group was injected at 0.1, 0.5, and 1 mg/mL in 10 mL of a solution containing about 120 Bq/g of Cs-137, and the sample was stirred and reacted for 24 hours to investigate the removal efficiency of radioactive cesium. After the experiment, the radiation dose of the sample was measured using analysis equipment provided by HPGe Gamma-ray Spectroscopy System. Adsorption experiments were conducted to evaluate the ability of the MAA-PAC-PB LBL group to remove radioactivity cesium (Cs-137) in water, and the results are shown in Fig. 8. The removal efficiency (R) and decontamination factor (DF) of Cs-137 were evaluated by the following Eqs (3) and (4).

3 $${\rm{Removal}}\,{\rm{efficiency}}\,( \% )=\frac{({A}_{0}-{A}_{f})}{{A}_{f}}X100$$

4 $${\rm{DF}}={A}_{0}/{A}_{f}$$

**Figure 9:**
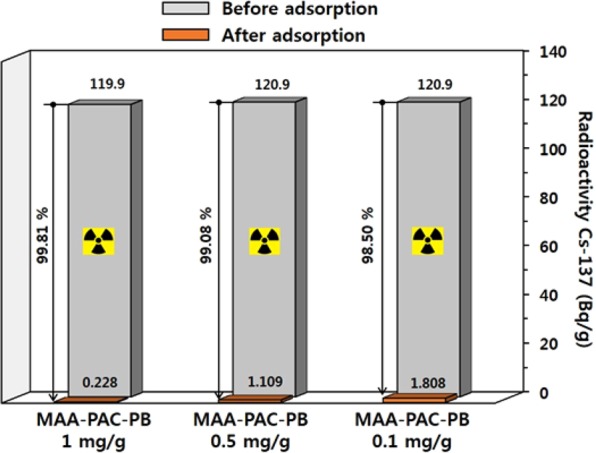
Radioactive cesium removal efficiency with increasing dose of MAA-PAC-PB LBL group.

As a result, when the adsorbent was injected at 0.1 mg/mL, cesium was removed by 98.5% from 120.9 Bq/q, which is an initial value, to 1.808 Bq/g. When the adsorbent was injected at 0.5 mg/mL, cesium was removed by 99.08% from 120.9 Bq/q, which is an initial value, to 1.109 Bq/g. In addition, when the adsorbent was injected at 1 mg/g, the initial solution concentration was 119.9 Bq/g and the concentration of the solution after the adsorption process was 0.228 Bq/g, which indicates a removal efficiency of 99.81% (Fig. 9). Furthermore, the decontamination factors (DF) according to the amount of injected adsorbents were 66.87, 109.02, and 525.88, respectively (Table 4). This confirms the potential application of in the MAA-PAC-PB LBL group, which exhibits high removal efficiency and DF, for decontamination of water that contains radioactive cesium. LBL group of MAA-PAC-PB developed in this study is synthesized by a relatively simple step. In addition, the reagents and synthetic materials used in the synthesis step are economically advantageous using domestic products. As mentioned above, adsorption is known as a way to efficiently treat radioactive cesium contaminated water without additional processes or facilities. Therefore, it can be used as an economical and effective method for removing radioactive cesium contaminated water through direct injection of LBL group of MAA-PAC-PB in existing water treatment facilities. Currently, the existing water treatment process has a facility to injection of PAC. Using this system, it can be easily injected MAA-PAC-PB LBL group for decontamination of radioactive cesium in case of an emergency.

**Table 4:** Cs-137 removal performance of MAA-PAC-PB.

MAA-PAC-PB LBL group (mg/g)	C-137 activity (Bq/g)		Performance	
	Initial	Final	R (%)	DF
0.1	120.9	1.808	98.50	66.87
0.5	120.9	1.109	99.08	109.02
1	119.9	0.228	99.81	525.88

## Conclusions

In summary, we have successfully developed economic and efficient Prussian blue based adsorbent immobilized in support particles to remove radioactive cesium (Cs-137). First, a hydrogel, which is formed through the polymerization of acrylic acid (AA), was synthesized. In order to improve the mechanical strength of the hydrogel, powder activated carbon (PAC) was impregnated. The synthesis of the PB was performed by using an *in-situ* method. The Fe^3+^ ion was preferentially adsorbed using the carboxyl group, which is the functional group of AA. After that, the PB was immobilized by reacting with ferrocyanide ion. In addition, Fe^3+^ ions were adsorbed through the LBL assembly process to supply again, through which the stable immobilization of the PB and the increase of the content in the adsorbent through the growth of PB particles were promoted. FT-IR and XRD analyses were performed to confirm the changes in the PB contents in the adsorbent by the LBL assembly synthesis method during the PB immobilization. As a result, it was concluded that the PB immobilization was successfully performed in both non-LBL and LBL groups. In addition, the SEM (EDS) analysis confirmed the improvement of Fe and K ion contents during the LBL assembly process, and the growth of PB particles was visually confirmed by the SEM image. After that, the surface and composition characteristics of the adsorbent material, which was synthesized through the TEM (EDS and mapping) analysis, were further confirmed. Furthermore, there was no leakage of the PB in the adsorbent material after the PB synthesis of the LBL group. Based on these results, we confirmed the stability of the PB immobilization was improved through the LBL assembly method, which is one of the PB immobilization strategies. As a result, it was concluded that the secondary environmental pollution due to the leakage of the adsorbed PB particles of radioactive cesium would not appear when the MAA-PAC-PB is applied in the real environment. The maximum adsorption amount of the MAA-PAC-PB LBL group on cesium was 40.03 mg/g. It was measured by using two isothermal adsorption models (Langmuir and Freundlich) and consequently, it was concluded that it would be more suitable for the Langmuir adsorption model. In order to find the removal efficiency for radioactive cesium (Cs-137) in the MAA-PAC-PB LBL group, the adsorbent was injected in a solution that contains about 120 Bq/g of Cs-137 by 0.1, 0.5, and 1 mg/mL. After the solution was reacted for 24 hours, the adsorbent and Cs solution were separated through solid-liquid separation. The final residual Cs-137 was 1.808, 1.109, and 0.228 Bq/g, respectively. The removal rates were 98.5, 99.08 and 99.81% of the initial radiation dose, respectively. In addition, high DF (>20) values were obtained under all adsorbent injection conditions, and it was concluded that the MAA-PAC-PB LBL group could be effectively applied to the treatment process for decontamination of radioactive cesium-contaminated water. The developed MAA-PAC-PB can be used to concentrate radioactive cesium in small volume and weight from the contaminated water, which makes it easy to handle for further radioactive waste disposal.

## Methods

### Materials

To Synthesized MAA-PAC, the following reagents were used: Powder activated carbon (SAMCHUN, KOREA), Acrylic acid (DAEJUNG, C_3_H_4_O_2_, 99%), sodium hydroxide (DAEJUNG, NaOH, 97%), potassium persulfate (DAEJUNG, K_2_S_2_O_8_, 98%), N,N’-methylenebisacrylamide (Sigma-aldrich, (CH_2_=CHCONH)_2_CH_2_), To Synthesized MAA-PAC-PB, Iron(III) chloride (SAMCHUN, FeCl_3_, 97%), Potassiumferrocyanide (SAMCHUN,K_4_Fe(CN)_6_·3H_2_O), CsCl (SAMCHUN, 99%), solution were reacted in order *in-situ*, and Radioactivitycesium(Cs-137) was obtained from a Satandard source solution certified by the Korea Research Institute of Standards and Science(KRISS).

### Optimization of MAA-PAC synthesis

In this study, the hydrogel was synthesized by impregnating powder activated carbon (PAC) to improve the mechanical strength of the hydrogel that was synthesized through polymerization of acrylic acid (AA). The synthesis procedure of the synthesized hydrogel is as follows. As a first solution, a mixed solution of 60 mg of PAC and 20 mL of tertiary distilled water was evenly dispersed by sonication for 90 minutes. As the second solution, 7.2 mL of AA and 10 mL of NaOH solution(0.01~2 M) were mixed in an ice water bath, the temperature of the solution was fixed at 5 °C or less, and the mixture was stirred. After that, silicone oil was injected into the tempered glass container and the temperature was maintained at 50 °C. Then, the first solution, which is the mixed solution of PAC and distilled water, and the second solution, which is a mixed solution of AA and sodium hydroxide, were simultaneously injected and stirred in nitrogen purged three-neck flask. After 1 hour, 80 mg of potassium persulfate (KPS) and 16 mg of MBA (N,N’-methylenebisacrylamide) were sequentially injected, the temperature of the mixture was increased to 70 °C, and the mixture was stirred. After 1 hour, 12 mL of NaOH (3 M) solution was additionally added, and it was further stirred at 70 °C for 1 hour. Finally, the synthesized hydrogel was washed with tertiary distilled water so that the pH becomes near neutral. After that, it was completely dried in a dry oven at a temperature of 60 °C and pulverized. Finally, the MAA-PAC was synthesized by filtering with 100 mesh.

### Synthesis of MAA-PAC-PB(non-LBL and LBL group)

0.1 g of the synthesized MAA-PAC was added to a 10 mM 50 mL solution of iron(III) chloride, and the mixture was stirred for 24 hours. After this, the MAA-PAC, in which Fe^3+^ ions were adsorbed through solid-liquid separation, was injected again to 10 mM 50 mM potassium ferrocyanide solution and stirred for 10 minutes. The Prussian blue was synthesized using the *in-situ* method for the first time. Then, in order to increase the content of Prussian blue, the adsorbent was added to a 10 mM 50 mL solution of iron(III) chloride, and the mixture was stirred for 10 minutes. The LBL-pretreated group of the synthesized MAA-PAC was synthesized. The non-LBL-pretreated group was synthesized only by up to the potassium ferrocyanide reaction.

### Effect of LBL assembly on stable PB synthesis

In order to analyze the surface characteristics of the PAC, MAA-PAC, and MAA-PAC-PB, transmission electron microscope (TEM, JEOL, JEM-2010, Japan) analysis was conducted, and energy dispersive spectroscopy (EDS) was additionally conducted to check the content of the elements that constitute each product and to determine the presence of synthesized PB in the MAA-PAC particles and the immobilization of the PB. Similarly, the XRD (Rigaku, SmartLab, Japan) and FT-IR (Thermo, Nicolet is 50) analyses were conducted at room temperature to verify the surface properties of the products at each synthesis step, and the spectrum ranged from 15 to 75 degrees and 500 to 3,000 cm^−1^. This characterization was further performed to change the PB content of the non-LBL and LBL groups of the MAA-PAC-PB. To confirm the outflow of the PB that was synthesized through non-LBL and LBL assembly processes in the MAA-PAC particles, 10 mg of the MAA-PAC-PB in each modified group was injected into 100 mL of distilled water and stirred for 2 hours. After 2 hours, sampling was conducted through solid-liquid separation, and 100 mL of fresh distilled water was injected and stirred. This process was repeated five times. The outflow of the PB was confirmed by UV spectrum (Biochrom, Libara S22, USA).

### Cesium adsorption performance test using Isotope(Cs-133)

Isothermal adsorption experiments were conducted at room temperature using a polypropylene falcon tube (50 mL). The samples were diluted to 0.1, 0.5, 5, 10, 100, 200, 600, and 1,000 mg/L (ppm) after a stock solution of 1,000 mg L^−1^ was prepare using CsCl reagent. After that, 0.01 g of the MAA-PAC-PB was added to each reaction solution, and solid-liquid separation was performed using a syringe filter after the solution was stirred for 24 hours. Samples were analyzed using ICP-MS (Perkin-Elmer, Nexion 350D, USA). In order to evaluate selective adsorption efficiency of the MAA-PAC-PB LBL group on Cs, adsorption experiments were conducted in solutions that contain competitive ions such as K^+^, Na^+^, Ca^2+^, and Mg^2+^. 100 mg/L of Cs and 3,000 ppm of competitive ions were prepared and reacted with 0.01 g of MAA-PAC-PB. In addition, artificial seawater (Na^+^ 10,000 ppm, Mg^2+^ 1,200 ppm, Ca^2+^ 600 ppm, K^+^ 500 ppm) was prepared to observe the change of Cs removal performance.

### Decontamination of radioactivity cesium(Cs-137)

Adsorption experiments were performed to evaluate the ability of MAA-PAC-PB to remove radioactivity cesium (Cs-137). The results are shown in Table 4. 1 mg/mL of the MAA-PAC-PB was added to 10 mL of the solutions that contain 1, 5, 10, and 100 Bq/g of Cs-137, and the mixtures were reacted for 24 hours. Furthermore, concentrations of Cs-137 in the solutions were measured for 3,600 seconds by using HPGe Gamma-ray Spectroscopy System.(CANBERRA, RAD Small Anode Germanium Well Detector, USA).

## Supplementary information

Supplementary Materials
