# Supplementary material for: Enhanced immobilization of Prussian blue through hydrogel formation by polymerization of acrylic acid for radioactive cesium adsorption
Source: Sci Rep. 2019 Nov 8;9:16334. doi: 10.1038/s41598-019-52600-z (PMC6841998; doi:10.1038/s41598-019-52600-z)
Supplement: Supplementary file 1 — Supplementary Materials [file 41598_2019_52600_MOESM1_ESM.docx]

**Enhanced immobilization of Prussian blue through hydrogel formation by polymerization of acrylic acid for radioactive cesium adsorption**

Daemin Oh^1^, Bokseong Kim^1^, Sungwon Kang^1,*^, Youngsug Kim^1^, Yoonshun Chung^1^,Sungjong Yoo^1^, Sol Kim^1^, Sungwook Choung^2^, Jeonghee Han^2^, Sunghee Jung^3^, Hyowon Kim^4^, Yuhoon Hwang^4,*^


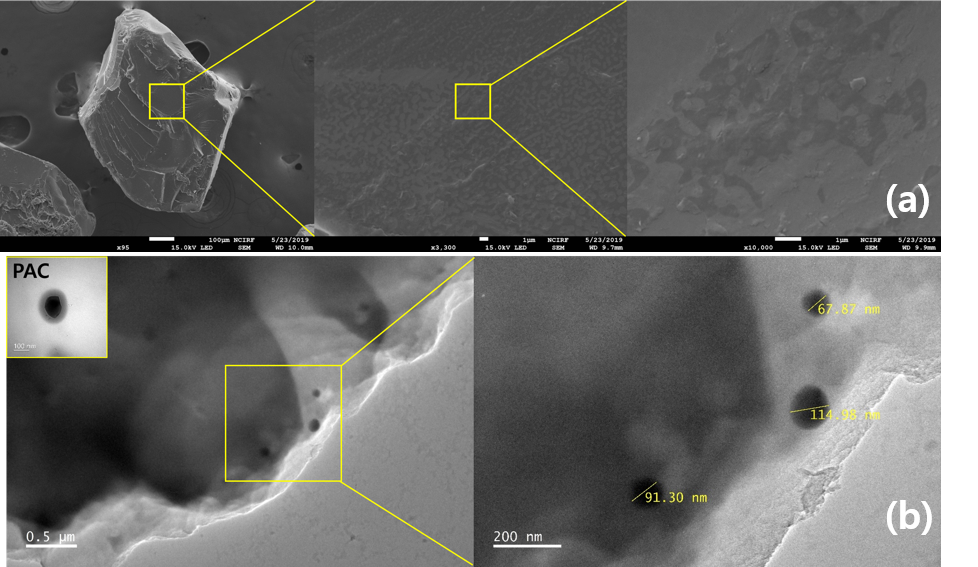


**Figure S1.** Morphology of MAA-PAC, (a) SEM image, (b) TEM image

**Table S1.** SEM-EDS analysis results of MAA-PAC, MAA-PAC after reaction with Fe^3+^

| **Items** | **MAA-PAC** | | **MAA-PAC + FeCl_3_** | |
| --- | --- | --- | --- | --- |
| **Elements** | **Weight %** | **Atomic %** | **Weight %** | **Atomic %** |
| C | 97.23 | 97.99 | 38.66 | 38.66 |
| O | 2.77 | 2.01 | 28.11 | 28.11 |
| K | - | - | 0.11 | 0.11 |
| Fe | - | - | 33.12 | 33.12 |
| Totals | 100 |  | 100 |  |

**Table S2.** TEM-EDS analysis results of MAA-PAC-PB(non-LBL group), MAA-PAC-PB(LBL group)

| **Items** | **Non-LBL group** | | **LBL group** | |
| --- | --- | --- | --- | --- |
| **Elements** | **Weight %** | **Atomic %** | **Weight %** | **Atomic %** |
| C | 55.34 | 71.82 | 40.88 | 61.87 |
| O | 22.47 | 21.90 | 22.92 | 26.05 |
| K | 0.77 | 0.31 | 2.24 | 1.04 |
| Fe | 21.42 | 5.98 | 33.95 | 11.05 |
| Totals | 100 |  | 100 |  |


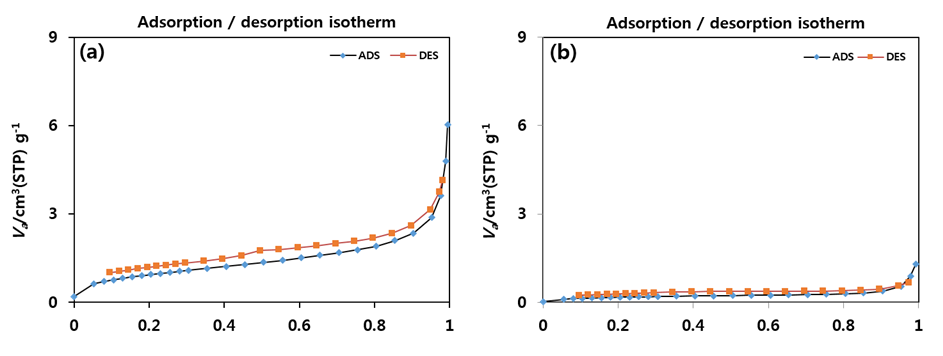


**Figure S2.** N_2_ gas adsorption(filled)-desorption(empty) isotherm of MAA-PAC-PB, (a) LBL group, (b) non-LBL group.

**Figure S3.** Cesium removal efficiency of MAA-PAC, MAA-PAC-PB LBL group, Prussian blue(4mM)


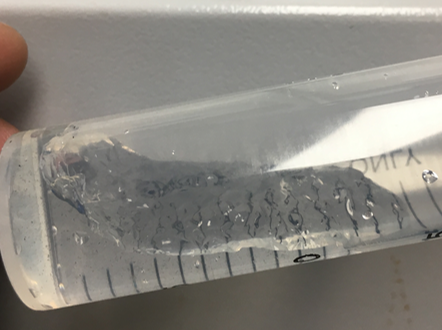


**Figure S4.** Hydrogelation of MAA-PAC in aqueous solution

**Table S3.** Reported maximum cesium adsorption capacities of PB based composites

| **Adsorbents** | **Langmuir isotherm** | | **Ref** |
| --- | --- | --- | --- |
|  | **q_m_(mg/g)** | **a_L_(L/mg)** |  |
| PB/Fe_3_O_4_/GO + Alginate (bead) | 43.5 | 0.219 | [1] |
| CNT/diatomite/PB + PU (sponge) | 2.94 | 0.884 | [2] |
| PB/ Fe_3_O_4_/GO + Pectin (powder) | 424 | 0.05 | [3] |
| PB/Graphene/Carbon fiber (fiber) | 81.23 | 0.094 | [4] |
| i-HIPE Hydrogel + PB (sponge) | 0.154 | 0.045 | [5] |
| PVA/alginate + PB (bead) | 3.33 | - | [6] |
| Cellulose nanofiber/PB + PVA (sponge) | 28.4 |  | [7] |
| Polyacrylonitrile nanofiber + PB (fiber filter) | 18.6 |  | [8] |
| MAA-PAC-PB (powder) | 40.0 | 0.012 | In this study |

**Table S4.** After the adsorption process, the concentration change of K^+^ ion

| **Items** | **Cs^+^** | **K^+^** |
| --- | --- | --- |
| Initial Conc. (mg/L) | 100.05 | 407.5 |
| Final Conc. (mg/L) | 57.35 | 423.3 |
| Variation Conc. (mg/L) | (-) 42.65 | (+) 15.8 |

**References**

[1] Yang H., Li H., Zhai J., Sun L., Zhao Y., and Yu H., "Magnetic prussian blue/graphene oxide nanocomposites caged in calcium alginate microbeads for elimination of cesium ions from water and soil," *Chem. Eng. J.,* **246**, 10-19(2014).

[2] Hu B., Fugetsu B., Yu H., and Abe Y., "Prussian blue caged in spongiform adsorbents using diatomite and carbon nanotubes for elimination of cesium," *Journal of Hazardous Materials,* **217-218**, 85-91(2012).

[3] Kadam A. A., Jang J., and Lee D. S., "Facile synthesis of pectin-stabilized magnetic graphene oxide prussian blue nanocomposites for selective cesium removal from aqueous solution," *Bioresource Technology,* **216**, 391-398(2016).

[4] Chen F., Jin G., Peng S., Liu X., and Tian J., "Recovery of cesium from residual salt lake brine in qarham playa of qaidam basin with prussian blue functionalized graphene/carbon fibers composite," *Colloids and Surfaces A: Physicochemical and Engineering Aspects,* **509**, 359-366(2016).

[5] Kim Y., Kim I., Lee T. S., Lee E., and Lee K. J., "Porous hydrogel containing prussian blue nanoparticles for effective cesium ion adsorption in aqueous media," *Journal of Industrial and Engineering Chemistry,* **60**, 465-474(2018).

[6] Lai Y., Chang Y., Chen M., Lo Y., Lai J., and Lee D., "Poly(vinyl alcohol) and alginate cross-linked matrix with immobilized prussian blue and ion exchange resin for cesium removal from waters," *Bioresource Technology,* **214**, 192-198(2016)

[7] Vipin, A. K., Fugetsu, B., Sakata, I., Isogai, A., Endo, M., Li, M., Dresselhaus, M. S., “Cellulose nanofiber backboned Prussian blue nanoparticles as powerful adsorbents for the selective elimination of radioactive cesium,” *Scientific Reports* **6**, 37009 (2016)

[8] Kim, H., Kim, M., Lee, W. & Kim, S. Rapid removal of radioactive cesium by polyacrylonitrile nanofibers containing Prussian blue. Journal of Hazardous Materials 347, 106-113
